# Ferritinophagic Flux Was a Driving Force in Determination of Status of EMT, Ferroptosis, and NDRG1 Activation in Action of Mechanism of 2-Pyridylhydrazone Dithiocarbamate S-Acetic Acid

**DOI:** 10.1155/2021/3015710

**Published:** 2021-12-07

**Authors:** Hao Li, Wei Zhou, Huiping Wei, Longlong Li, Xu Wang, Yongli Li, Shaoshan Li, Changzheng Li

**Affiliations:** ^1^Department of Surgery, The Third Affiliated Hospital of Xinxiang Medical University, Xinxiang, Henan 453003, China; ^2^Department of Histology and Embryology, Sanquan College of Xinxiang Medical University, Xinxiang, Henan 453003, China; ^3^College of Pharmacy, Sanquan College of Xinxiang Medical University, Xinxiang, Henan 453003, China; ^4^College of Basic Medical Science, Xinxiang Medical University, Xinxiang, Henan 453003, China

## Abstract

Ferritinophagy is a process of ferritin degradation in lysosomes; however, how its effect on other cellular events, such as epithelial-mesenchymal transition (EMT) and ferroptosis remains elusive. In this study, we determined how ferritinophagic flux influence the status of EMT and ferroptosis in HepG2 cell. Our data revealed that 2-pyridylhydrazone dithiocarbamate s-acetic acid (PdtaA) induced EMT inhibition involved ferritinophagy-mediated ROS production, but addition of ferrostatin-1 could attenuate the effect of PdtaA on the regulation of EMT-related proteins, suggesting that ferroptosis might involve in the EMT regulation. Next, downregulation of Gpx4 and xCT as well as enhanced lipid peroxidation further supported that PdtaA was able to induce ferroptosis. Knockdown of NCOA4 significantly attenuated the regulatory effect of PdtaA on related proteins which highlighted that the strength of ferritinophagic flux (NCOA4/ferritin) was a driving force in determination of the status of EMT and ferroptosis. Furthermore, NDRG1 activation was also observed, and knockdown of NDRG1 similarly influenced the expressions of ferroptosis-related proteins, suggesting that NDRG1 also involved ferroptosis induction, which was first reported. Taken together, PdtaA-induced EMT inhibition, ferroptosis, and NDRG1 activation all depended on the strength of ferritinophagic flux.

## 1. Introduction

Hepatocellular carcinoma is the third leading cause of cancer death [1]; surgical resection, transplantation, ablation, transarterial chemoembolization [2, 3], and tyrosine-kinase inhibitors (sorafenib, lenvatinib, and regorafenib) are generally used treatments for this disease [4]. Clearly, chemotherapy is still an important clinical treatment; however, its side effects and drug resistance generated are commonly faced. To improve the therapeutic effect, therefore, a new therapeutic strategy for liver cancer therapy requires to be developed. Recently, the role of tumor microenvironment in tumor metastasis has been realized [5]; tamed macrophages (or called tumor-associated macrophages (TAMs)) in the tumor environment can phagocytize apoptotic or necrotic cells, as well as senescent blood cells, which releasing iron, copper ions, and cytokines for tumor cells growth and angiogenesis. Therefore, depletion of the metal ion by chelation was proposed to inhibit tumor cell growth [6, 7].

Epithelial-to-mesenchymal transition (EMT) is a cellular process wherein epithelial cells lose their junctional architecture, endowing the polarized epithelium cells a more invasive features [8, 9]; thus, cancer cell metastasis is considered to be related to EMT transition. Multiple molecular mechanisms were reported involving EMT transition, including growth factors and cytokines [10]. Transforming growth factor (TGF-*β*1) is commonly used in the induction of EMT. In addition, EMT transition in hepatocellular carcinoma can be regulated directly and indirectly through other genes, microRNA and LncRNA [11–14]; clearly, EMT regulation is complex and remains to be determined. Undoubtedly, insight into the mechanism of EMT would help us to develop a new strategy or inhibitor in order to inhibit EMT and cancer metastases.

Mitochondria are the main sites for reactive oxygen species (ROS) production, and the species include superoxide anion and hydrogen peroxide [15]. In addition, hydroxyl radical is one of species of ROS, which is generated from hydrogen peroxide reaction with iron released from degradation of iron-containing proteins in lysosomes and proteasomes [16, 17]. Therefore, ROS as an umbrella term covers a range of small molecule oxidizing, nitrosating, nitrating, halogenating, and thiol-reactive species, produced in biological systems [18].

Ferritin degradation in lysosomes requires the assistance of NCOA4, and the process was termed ferritinophagy [19], which also generates ROS, especially hydroxyl radical. Some iron chelators act as ferritinophagy inducers, but if the occurrence of ferritinophagy resulting in ROS production is pluralistic. DFO can induce ferritinophagy, but often is used as a ROS scavenger, while dithiocarbamate derivatives act both in ferritinophagy and ROS inducer [20, 21]. Ferroptosis is an iron-dependent cell death that is executed by reactive oxygen species (ROS)-mediated peroxidation of polyunsaturated fatty acids (PUFAs). Lipid peroxidation can be reduced by glutathione peroxidase 4 (GPx4)-mediated glutathione (GSH) [22]. Cysteine is a material for GSH synthesis, derived from cystine reduction. Cystine is transported into the cell through the system Xc^−^ transporter through SLC7A11 subunit. Therefore, GPx4 and Xc^−^ system modulate cellular redox homeostasis and determine ferroptosis fate [23].

N-myc downstream regulated gene 1 (NDRG1) belongs to the NDRG protein family consisting of NDRG1-4, which are evolutionarily well conserved and predominantly localized in the cytoplasm [24]. NDRG1 has contradictory roles in cancers, either suppressing metastasis and oncogenesis or promoting tumor growth and metastasis [24]. It was reported that a marked enhancement of NDRG1 expression resulted in tumor growth suppression [25]; in addition, NDRG1 as a stress responder has been shown to associate with EMT [26]. 2-Pyridylhydrazone dithiocarbamate s-acetic acid (PdtaA) exhibited certain antitumor activity against HepG2 in our previous work [27], with insight into more molecular details in the action of the mechanism of the agent; in the present study, we investigated the effect of PdtaA on EMT regulation, revealing that PdtaA-induced EMT inhibition involved ROS production that derived partly from ferritinophagy induction. While, the alterations in ferroptosis-related proteins implied PdtaA being able to induce ferroptosis. In addition, NDRG1 was activated and involved in regulation of the ferroptosis, which was report for the first time. The correlation analysis revealed that PdtaA induced EMT inhibition, ferroptosis induction, and NDRG1 activation, all depended on the strength of ferritinophagic flux (ratio of NCOA4/ferritin).

## 2. Results

### 2.1. PdtaA-Induced EMT Inhibition Involved ROS Production

**Figure 1:**
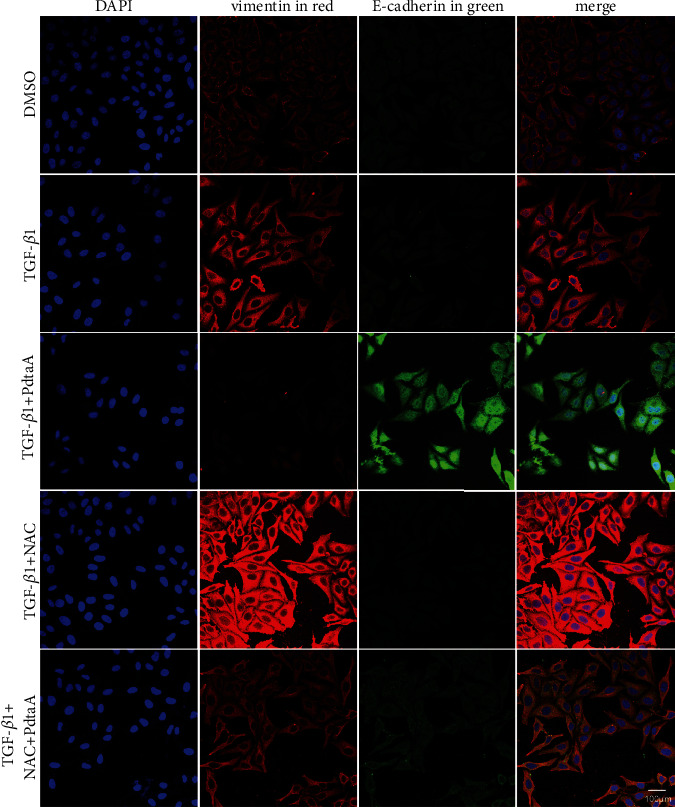
PdtaA-induced EMT inhibition involved ROS production. The dose of PdtaA used in all experiments was at 25 *μ*M, unless otherwise specified. Objective size: 40 × 10, scale bar: 100 *μ*m.

An alteration in the morphology of HepG2 cells was noted when the cells are exposed to PdtaA (Fig S1), which prompted us to think about whether the morphology change involved EMT regulation. Thus, an EMT model was first established through TGF-*β*1 induction. The levels of EMT-related proteins (E-cadherin and vimentin) before and after PdtaA treatment were determined by the immunofluorescence method.  Figure 1 shows that TGF-*β*1 treatment led to a significant increase of vimentin compared to that of nontreatment; however, addition of PdtaA could cripple the regulatory effect of TGF-*β*1 on EMT regulation, i.e., vimentin was weakened in color intensity, while E-cadherin accordingly markedly enhanced (Figure 1) based on the alterations in colors. In addition, ROS production was reported in the EMT process [27, 28], if a similar situation occurred in PdtaA induced EMT inhibition? To this end, N-acetyl-L-cysteine (NAC), a ROS scavenger was used to determine if ROS production involved the EMT inhibition. Thus, we cotreated the HepG2 cells with PdtaA and NAC.  Figure 1 clearly shows that the combined treatment indeed weakened the regulatory effect of PdtaA on EMT regulation, suggesting that ROS production was involved in the EMT inhibition.

### 2.2. PdtaA-Induced EMT Inhibition Involved Autophagy

**Figure 2:**
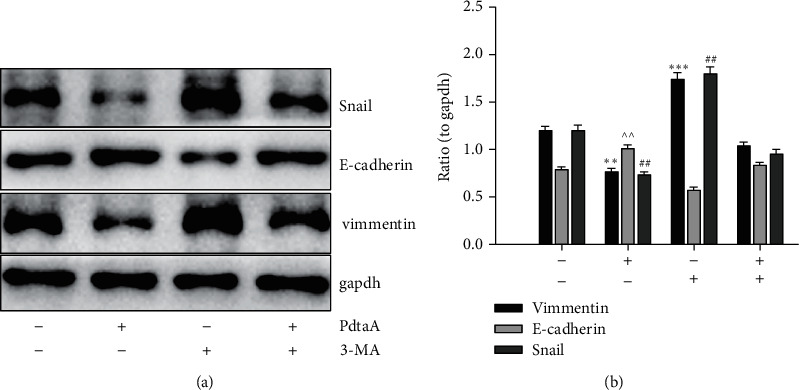
PdtaA-induced EMT inhibition involved autophagy. (a) Western blotting analysis of EMT-related proteins. (b) Quantitative analysis derived from (a). The quantitative analysis of EMT-related proteins is from twice experiments (^*∗∗*^^, ^^, ##^*p* < 0.05; ^*∗∗∗*^*p* < 0.01 vs. control, one-way ANOVA with Dunnett post hoc correction).

It has been showed that autophagy is accompanied by the production of ROS [29], if this occurred in our study. For this purpose, an autophagy inhibitor, 3-methyladenin (3-MA), was further used to determine whether autophagy involved PdtaA-induced EMT inhibition.  Figure 2 shows that addition of 3-MA markedly attenuated the regulatory effect of PdtaA on EMT-related proteins, implying that the EMT inhibition involved autophagy. The additional of information from Figure 2 shows that the EMT inhibition induced by PdtaA owed to autophagic degradation of Snail, an EMT transcription factor, which led to downregulation of vimentin.

### 2.3. PdtaA-Induced Ferritinophagy Was Redox Active and Partly Responsible for the ROS Production

**Figure 3:**
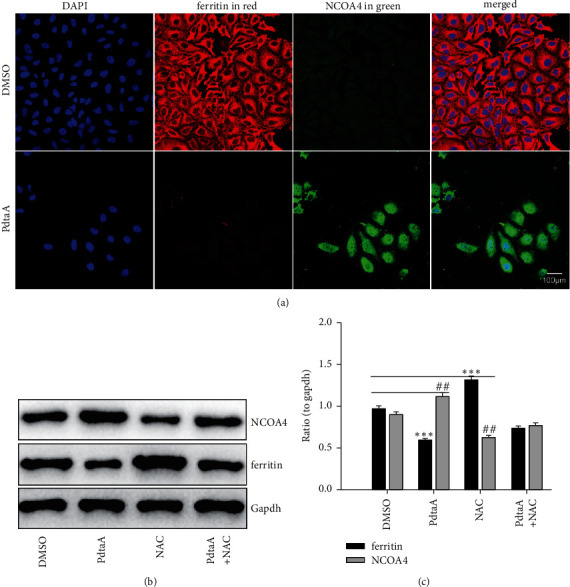
PdtaA-induced ferritinophagy was responsible for ROS production. (a) Immunofluorescence analysis. (b) Western blotting analysis. (c) Quantitative analysis derived from (b). The results from (c) are from twice experiments (^*∗∗*^^, ##^*p* < 0.05; ^*∗∗∗*^*p* < 0.01 vs. control, one-way ANOVA with Dunnett post hoc correction).

Now that ROS production involved EMT inhibition induced by PdtaA, the source of ROS production required to be determined. Mitochondria were the main source of ROS; in addition, degradation of iron-containing protein in proteasomes and lysosomes also contributes to ROS production. NCOA4-mediated ferritin degradation in lysosomes (ferritinophagy) was observed in our previous investigation [21]; similar scene might occur in action of the mechanism of PdtaA. To this end, the levels of ferritin and NCOA4 were assayed via the immunofluorescence technique. As shown in Figure 3(a), a decrease of red fluorescence of ferritin and an enhanced green fluorescence of NCOA4 were observed after PdtaA treatment. The merged photos showed clearly the changes, indicating that the ferritin degradation was through autophagic degradation. Additional results from Western blotting analysis further supported above conclusion because PdtaA-induced ferritin downregulated and NCOA4 upregulated (Figure 3(b)) and was in consistent with the result from immunofluorescence (Figure 3(a)). This clearly indicated that PdtaA was able to induce the occurrence of ferritinophagy.  Figure 3(c) shows that the differences of ferritinophagy-related proteins were significant in statistics (*p* < 0.05) before and after PdtaA treatment. Importantly, addition of NAC significantly attenuated the regulatory effect of PdtaA on ferritinophagy-related proteins, indicating that PdtaA-induced ferritinophagy was redox active and responsible for the ROS production.

### 2.4. NCOA4-Mediated Ferritinophagy Contributed to the EMT Inhibition

**Figure 4:**
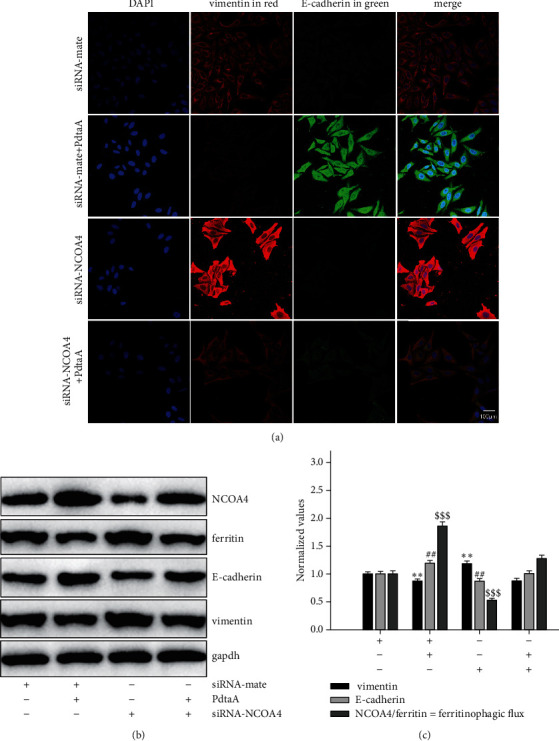
NCOA4 involved the EMT inhibition induced by PdtaA. (a) Immunofluorescence analysis of EMT-related proteins. Objective size: 40 × 10, scale bar: 100 *μ*m. (b) Alterations of EMT and ferritinophagy-related proteins. (c) Quantitative analysis derived from (b). The results from (c) are from twice experiments. (^*∗∗*^^, ##^*p* < 0.05; ^$$$^*p* < 0.01 vs. control, one-way ANOVA with Dunnett post hoc correction).

As mentioned above, PdtaA induced both ferritinophagy and EMT inhibition, the causal relationship between the two cellular events required to be further determined. To this end, NCOA4 was knocked down to determine its role in EMT regulation. As shown in Figure 4, knockdown of NCOA4 resulted in an obvious increase of vimentin compared to that in the siRNA-mate group. Although PdtaA treatment resulted in upregulation of E-cadherin and downregulation of vimentin (Figure 4(a)), the changes in EMT-related proteins were significantly attenuated by knockdown of NCOA4 (Figure 4(a)). Similar results were from Western blot analysis (Figure 4(b)). Those supported that there was a correlation between ferritinophay induction and EMT inhibition. To emphasize the role of ferritinophagy in the EMT process, the ratio of NCOA4 to ferritin in abundance was termed as ferritinophagic flux and used for indicative of the strength of ferritinophagy. Figure 4(b) clearly shows that the stronger ferritinophagic flux, the greater inhibition of EMT, indicating that the ferritinophagic flux was a dominant driving force in the determination of EMT state [22, 28]. The quantitative analysis from Western blotting is shown in Figure 4(c), and the differences in E-cadherin and vimentin before and after PdtaA treatment were significant (*p* < 0.05).

### 2.5. Ferroptosis Induction Was due to an Occurrence of Redox Active Ferritinophagy

**Figure 5:**
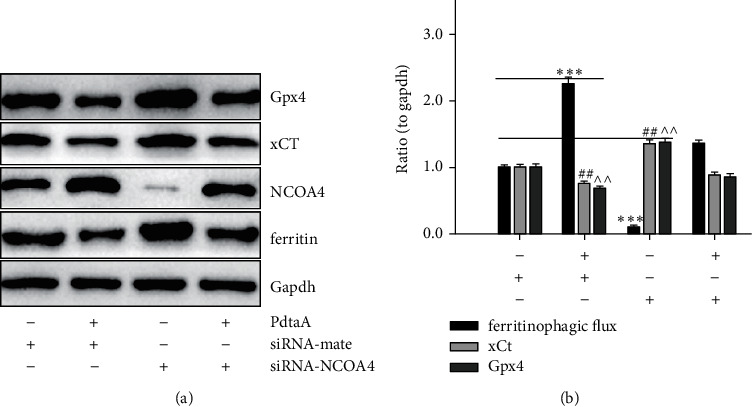
Enhanced ferritinophagic flux contributed to the ferroptosis induction. (a) The effect of knockdown of NCOA4 on ferritinophagy and ferroptosis-related proteins. (b) Quantitative analysis derived from (a). The data in quantitative analysis of related proteins in Figure 5(b) are from twice experiments (^^^, ##^*p* < 0.05; ^*∗∗∗*^*p* < 0.01 vs. control, one-way ANOVA with Dunnett post hoc correction).

Ferroptosis is an iron-dependent accumulation of reactive oxygen species (ROS) that leads to lipid peroxidation and cell death [30]; however, the causal relationship between ferritinophagy and ferroptosis was still lacking. PdtaA induced an occurrence of ferritinophagy, involving iron-mediated ROS production, required for ferroptosis induction. Gpx4 inhibition is a significant hallmark and indicator of ferroptosis [31, 32]. In agreement with these reports, the depletion of Gpx4 and xCT in our study (Figure S2) seemed to support that PdtaA had the ability of ferroptosis induction. To support the above conclusion that the depletions of Gpx4 and xCT correlated with the ferritinophagy induction, NCOA4 was genetically knocked down by small-interfering RNA. As shown in Figure 5(a), knockdown of NCOA4 resulted in increase in both Gpx4 and xCT and markedly attenuated the regulatory effect of PdtaA on ferroptosis-related proteins. The quantitative analysis clearly demonstrated that the ferritinophagic flux was a crucial factor in determination of the status of ferroptosis (Figure 5(b)), i.e., the stronger the ferritinophagic flux, the higher chance in ferroptosis induction. Similarly, on addition of an autophagy inhibitor, chloroquine could also achieve similar results, supporting that autophagy (ferritinophagy) played a role in ferroptosis induction (Figure S3). The enhanced lipid peroxidation after PdtaA treatment corroborated that PdtaA could induce ferroptosis (Figure S4).

### 2.6. The Regulatory Effect of Ferroptosis on EMT

**Figure 6:**
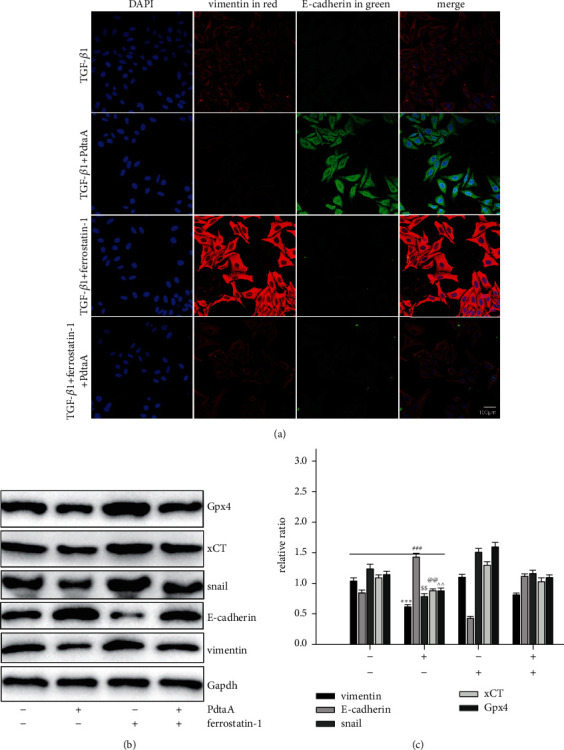
Ferroptosis involved PdtaA-induced EMT inhibition. (a) Immunofluorescence analysis of EMT-relative proteins. Objective size: 40 ×10, scale bar: 100 *μ*m. (b) Western blotting analysis of ferroptosis and EMT-related proteins. (c) Quantitative analysis derived from (b). The results from (c) are from trice experiments (^$$, @@, ^^^*p* < 0.05; ^*∗∗∗*^, ^###^*p* < 0.01 vs. control, one-way ANOVA with Dunnett post hoc correction).

Since PdtaA induced both ferroptosis and EMT inhibition, the interrelation between the two cellular events needed to be clarified. To this end, the cells that underwent EMT were treated by PdtaA, ferrostatin-1, and combination of them, respectively. The immunofluorescence results are shown in Figure 6(a). It was surprised that ferrostatin-1 treatment could upregulate vimentin and downregulate E-cadherin; thus, it could be imagined that the regulatory effect of PdtaA on EMT was neutralized. Western blot analysis in Figure 6(b) shows that ferrostatin-1 treatment resulted in a significant increase in Snail and a decrease in E-cadherin, but no obvious changes in those related proteins in the combination treatment. Those clearly indicated that ferroptosis induced by PdtaA had a certain role in EMT regulation. The quantitative analysis of relative proteins is shown in Figure 6(c); the difference in ferroptosis and EMT-related proteins before and after PdtaA treatment was significant in statistics (*p* < 0.05).

### 2.7. NDRG1 Activation Correlated with Ferroptosis Induction and EMT Inhibition

**Figure 7:**
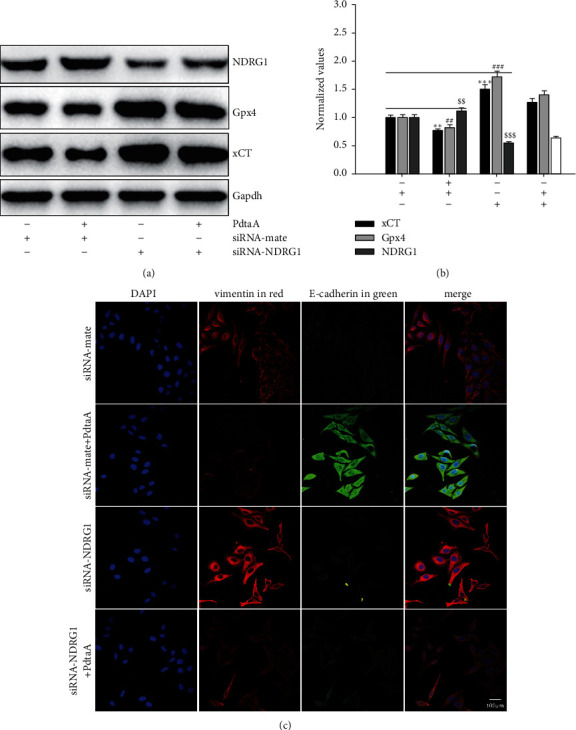
NDRG1 activation involved both ferroptosis and EMT regulation in action of mechanism of PdtaA. (a) The effect of NDRG1 on ferroptosis-related proteins. (b) Quantitative analysis derived from (a). The quantitative analysis of ferroptosis-related proteins is from twice experiments (^*∗∗*^^, $$, ##^*p* < 0.05; ^*∗∗∗*^^, ###, $$$^*p* < 0.01 vs. control, one-way ANOVA with Dunnett post hoc correction). (c) The effect of NDRG1 on EMT-related proteins. Objective size: 40 ×10, scale bar: 100 *μ*m.

NDRG1 protein has been associated with numerous cellular events, such as EMT and stress response [33–35]. Ferroptosis is a ROS-dependent cell death, which may trigger NDRG1 response. To this end, the level of NDRG1 was assayed. As expected (Figure S5), a significant increase of NDRG1 was observed upon PdtaA treatment. To insight into the association between ferroptosis and NDRG1, genetical knockdown of NDRG1 was performed.  Figure 7(a) shows that knockdown of NDRG1 led to upregulation of Gpx4 and xCT; thus, it is conceivable that NDRG1 knockdown would attenuate the regulatory effect of PdtaA on ferroptosis induction, indicating that NDRG1 also involved the ferroptosis induction. To our knowledge, the association between ferroptosis and NDRG1 was first reported. The quantitative analysis of those proteins is shown in Figure 7(b). In addition, the effects of NCOA4 and NDRG1 on lipid peroxidation were further investigated.  Figure S6 shows that knockdown of both NCOA4 and NDRG1 could attenuate the effect of PdtaA on lipid peroxidation, but the regulatory effect of NCOA4 was stronger than that of NDRG1; those indicated that both NCOA4 and NDRG1 were all involved in ferroptosis regulation.

In addition, NDRG1 activation associated with an iron chelator induced EMT inhibition [36]; this may occur in the mechanism of action of PdtaA due to its iron chelating ability. Thus, the effect of NDRG1 on EMT regulation in HepG2 cells was further investigated. As shown in Figure 7(c), knockdown of NDRG1 led to a slight increase in vimentin, supporting that NDRG1 involved EMT regulation, in consistent with the results reported previously [37].

## 3. Discussion

Vimentin and E-cadherin are important markers in epithelial-mesenchymal transition (EMT) process; thus, an increase in vimentin or a decrease in E-cadherin was considered the cells undergoing EMT [37, 38]. In cancer cells, E-cadherin is suppressed through the transcriptional repressors (SNAIL, SLUG, and ZEB1) binding to the E-cadherin promoter [39, 40]. Our data showed that PdtaA treatment caused a significant downregulation of vimentin and an upregulation of E-cadherin (Figures 1, 2), supporting that PdtaA was able to inhibit EMT. In addition, compelling evidence reveals reactive oxygen species (ROS) as crucial conspirators in EMT engagement [41], but whether EMT inhibition was achieved through massive ROS is unclear. In our study, addition of NAC significantly attenuated the regulatory effect of PdtaA on EMT-related proteins, clearly indicating that the EMT inhibition involved ROS production (Figure 1). This further strengthens the viewpoint that ROS have a dual role in EMT regulation, moderate ROS promotes EMT, and massive ROS production reverses EMT [42]. Next, the origin of ROS was further explored. Mitochondria are the main sites of ROS production, but lysosomes and proteasomes are also auxiliary sources [43]. It was reported that ferritin degradation contributed to EMT was reported [28], a similar observation was in the present study. Interestingly, we demonstrated that PdtaA similarly induced ferritinophagy as other dithiocarbamate acted (Figure 3) [28, 44], which at least, partly contributed to the ROS production [21, 22]. Next, the causal relationship between EMT and ferritinophagy was determined. We knocked down NCOA4 to observe its effect on EMT status; such action attenuated the regulatory effect of PdtaA on EMT inhibition in HepG2 cells, strengthening this concept that “ferritinophagic flux (NCOA4/ferritin)” was a dominant driving force in the determination of EMT status (Figure 4), which was in consistent with that reported previously [22, 28].

Ferroptosis is caused by iron-dependent ROS production [45]. Ferritinophagy may trigger ferroptosis accordingly [46]; however, ferritinophagy was not necessarily for ferroptosis, such as DFO-induced ferritinophagy, but the lack of ROS production is due to the formation of a nonredox activity chelate [47]. Thus, the causal relationship between ferritinophagy and ferroptosis still needed to be determined. The depletions of Gpx4, xCT, and cellular antioxidants as well as accumulation of lipid peroxidation are when HepG2 cells exposure to PdtaA supported that PdtaA acted as a ferroptosis inducer (Figure 5). Our data clearly illustrated that the stronger the ferritinophagic flux, the higher chance of ferroptosis induction (Figure 5(b)), indicating that ferritinophagy-mediated ROS production is a major requisite for ferroptosis induction. It was noted that although many compounds can trigger ferroptosis [32], iron chelator originated from dithiocarbamate derivatives as ferroptosis inducer was few.

**Figure 8:**
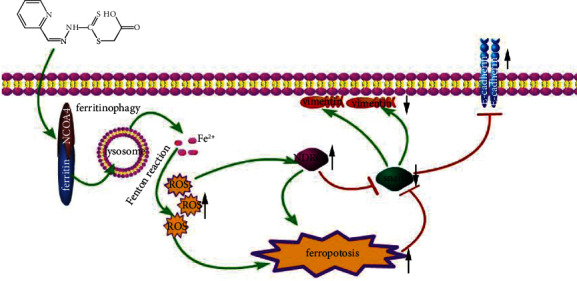
The action of mechanism of PdtaA in EMT inhibition. PdtaA treatment resulted in ferritinophagy that triggered ROS production. ROS production further triggered to ferroptosis and NDRG1 response (activation), which caused snail downregulation, and ultimately achieved the EMT inhibition (downregulation of vimentin and upregulation of E-cadherin).

Recently, a study showed that TGF-*β*1 could induce EMT and restrain ferroptosis [48]. PdtaA treatment resulted in EMT inhibition and ferroptosis induction, indicating that the agent had a reverse action compared to TGF-*β*1 (Figure 6). Therefore, PdtaA acted as EMT inhibitor. In addition, our data supported that EMT inhibition benefited from ferroptosis induction. On the other hand, NDRG1 as a stress responder associates with EMT [35, 49]. Similarly, NDRG1 activation was shown in iron chelators-induced EMT inhibition [50]. Our data demonstrated that NDRG1 activation was also associated with ferroptosis induction (Figure 7), while NDRG1 knockdown led to a significant increase in Gpx4 and xCT and further highlighted the concept that NDRG1 also regulated the ferroptosis process. Above all, the action of the mechanism of PdtaA in EMT inhibition could be proposed based on the presented data (Figure 8).

Taken together, PdtaA induced EMT inhibition, ferroptosis induction, and NDRG1 activation in HepG2 cell depending on its ability in ferritinophagy induction. However, the detail of how NDRG1 regulates ferroptosis was not fully solved and required more investigations. Similarly, more cell lines used to support the above conclusions are required in a future study.

## 4. Materials and Methods

### 4.1. General Information

HepG2 cell line was obtained from HonorGene (Changsha, China). Ferrostatin-1, MTT, 3-methyladenin (3-MA), chloroquine (CQ), RPMI-1640, and other chemicals were purchased from Sigma-Aldrich. 2-Pyridylhydrazone dithiocarbamate s-acetic acid (PdtaA) was prepared in our laboratory [27]. GPx4, xCT (SLC7A11), NDRG1, vimentin, and NCOA4 antibody were obtained from the Proteintech Group (Wuhan, China). Antibodies of E-cadherin and secondary antibodies (fluorescence-labeled for immunofluorescence) were purchased from Cell Signaling Technology (USA). Ferritin antibody for immunofluorescence was obtained from Santa Cruz Biotechnology (USA, Santa Cruz). NCOA4 antibody for immunofluorescence was purchased from Atlas Antibody (Sweden). Secondary antibodies for Western blotting were obtained from EarthOx, LLC (San Francisco, USA).

### 4.2. ROS Detection

3 × 10^5^ HepG2 cells were treated with given conditions (PdtaA or transfection) 24 h following PBS washing and trypsin digestion; finally, the cells were resuspended in H_2_DCF-DA containing serum-free culture medium and incubated for 30 min (active oxygen detection kit, Beyotime Biotechnology). The intracellular ROS was determined on a flow cytometer (CytoFLEX, Beckman Coulter, USA).

### 4.3. Immunofluorescence Analysis

The HepG2 cells were grown in 6-well plates with cover glass overnight. Following the indicated treatments (PdtaA or transfection), the cells were fixed with 4% paraformaldehyde, permeabilized with 0.2% triton-X-100, blocked with 1% BSA, and incubated with either ferritin (H chain, Santa Cruz Biotechnology) or combined with NCOA4 (Atlas antibodies) primary antibody at 4°C for overnight based on the protocol described previously [18]. Next, the cells were further incubated with fluorescence-labeled secondary antibody for 3 h at room temperature and were further counterstained with DAPI after removing the secondary antibody. Finally, visualization of the cells was on a confocal laser scanning microscope (Nikon Eclipse Ts2, Japan), and the representative cells were selected and photographed.

### 4.4. Knockdown of NCOA4 and NDRG1

The procedure as described previously for knockdown of NCOA4 and NDRG1 was followed [10]. The small-interfering RNA (siRNA-mate (siN0000001-1-5) and siRNA (siG000008031A-1-5, siG000008031B-1-5 for NCOA4; siB12314144804A-1-5, siB12314144746-1-5 for NDRG1) were obtained from RiboBio, China. Briefly, the HepG2 cells (1 × 10^6^) were transfected with 100 pmol of siRNA using Lipofectamine™ Stem Transfection Reagent (Invitrogen, USA) for 12 h. For immunofluorescence analysis, the HepG2 cells were first cultured in 24-well plates with cover glass overnight, and then, transfection was conducted as described above.

### 4.5. Western Blotting Analysis

HepG2 cells at given conditions were scraped off in lysis buffer and hydrolyzed on ice for 30 min., as described previously [10]. The supernatant was collected by centrifugation (14,000 × *g*) and stored at −80°C. Upon determination of the protein concentrations, 30 *μ*g of proteins was loaded on a 13% sodium dodecyl sulfate-polyacrylamide gel (SDS-PAGE) and subjected to electrophoresis and transferred onto a PVDF membrane. After blocked by 5% nonfat skimmed milk in TBS containing 0.1% Tween-20 for 2 h, the membrane was incubated at 4°C overnight with the primary monoantibody at a dilution of 1 : 300 in TBST buffer. Finally, the membrane was incubated with a secondary antibody (1 : 2,000 in TBST) for 1 h at room temperature. The protein bands were determined on an Syngene G: BOX Chemi XX9 (Syngene, UK).

### 4.6. Statistical Analysis

Results are presented as the mean ± SEM. The comparisons between multiple groups were performed by one-way ANOVA with Dunnett post hoc correction. A *P* value < 0.05 was considered statistically significant.

## Data Availability

The data used to support this study are included within the article.

## Supplementary Materials

Supplementary Materials Figure S1. The effect of PdtaA on morphology of HepG2 cell. (a) DMSO (70%). (b) PdtaA. Arrowheads are the cells that underwent morphologic change. Objective size: 20 ×10. Figure S2. PdtaA induced a downregulation of ferroptosis-related proteins. (a) Western blotting analysis and (b) quantitative analysis derived from (*A*). ^##,^^*∗∗*^*P* < 0.05; ^*∗∗∗*^^, ###^*p* < 0.01 vs. control. Figure S3. Chloroquine attenuated the action of PdtaA on the regulatory effect on ferroptosis-related proteins. (a) Western blotting analysis and (b) quantitative analysis derived from (*A*). ^##^, ^*∗∗*^, ^$$^*P* < 0.05; ^*∗∗∗*^*p* < 0.01 vs. control. Figure S4. PdtaA treatment results in alteration in lipid peroxidation. ^*∗∗∗*^^, ###^*P* < 0.01 vs. control. Figure S5. PdtaA treatment results in an upregulation of NDRG1. (a) Western blotting analysis and (b) quantitative analysis derived from (*A*). ^*∗∗*^*P* < 0.05; ^*∗∗∗*^*p* < 0.01 vs. control. Figure S6. Activation of NCOA4 and NDRG1 involved PdtaA-induced ferroptosis. The HepG2 cells treated with either siRNA-mate or siRNA-NCOA4, followed by PdtaA treatment. The lipid peroxidation was measured based on the method described previously. The molar absorptivity of the ferric thiocyanate complex expressed per mol of LOOH was determined to be 58,440 M^−1^ cm^−1^ [2]. ^##^*P* < 0.05 vs. control; ^*∗∗∗*^*p* < 0.01 vs. control.

## References

[1] Forner A., Reig M., Bruix J. (2018). Hepatocellular carcinoma. *The Lancet*.

[2] Llovet J. M., Real M. I., Montaña X. (2002). Arterial embolisation or chemoembolisation versus symptomatic treatment in patients with unresectable hepatocellular carcinoma: a randomised controlled trial. *The Lancet*.

[3] Lo C.-M., Ngan H., Tso W.-K. (2002). Randomized controlled trial of transarterial lipiodol chemoembolization for unresectable hepatocellular carcinoma. *Hepatology*.

[4] Cheng H., Sun G., Chen H. (2019). Trends in the treatment of advanced hepatocellular carcinoma: immune checkpoint blockade immunotherapy and related combination therapies. *American Journal of Cancer Research*.

[5] Sounni N. E., Noel A. (2013). Targeting the tumor microenvironment for cancer therapy. *Clinical Chemistry*.

[6] Khan G., Merajver S. (2009). Copper chelation in cancer therapy using tetrathiomolybdate: an evolving paradigm. *Expert Opinion on Investigational Drugs*.

[7] Bogaard H. J., Mizuno S., Guignabert C. (2012). Copper dependence of angioproliferation in pulmonary arterial hypertension in rats and humans. *American Journal of Respiratory Cell and Molecular Biology*.

[8] Savagner P. (2015). Epithelial-mesenchymal transitions. *Current Topics in Developmental Biology*.

[9] Lamouille S., Xu J., Derynck R. (2014). Molecular mechanisms of epithelial-mesenchymal transition. *Nature Reviews Molecular Cell Biology*.

[10] Odero-Marah V., Hawsawi O., Henderson V., Sweeney J. (2018). Epithelial-mesenchymal transition (EMT) and prostate cancer. *Advances in Experimental Medicine & Biology*.

[11] Zhang G., Zhang G. (2019). Upregulation of FoxP4 in HCC promotes migration and invasion through regulation of EMT. *Oncology letters*.

[12] Guo D., Li Y., Chen Y. (2019). DANCR promotes HCC progression and regulates EMT by sponging miR-27a-3p via ROCK1/LIMK1/COFILIN1 pathway. *Cell Proliferation*.

[13] Wang J., He H., Jiang Q., Wang Y., Jia S. (2020). CBX6 promotes HCC metastasis via transcription factors snail/zeb1-mediated EMT mechanism. *OncoTargets and Therapy*.

[14] Zhou Y., Huan L., Wu Y. (2020). LncRNA ID2-AS1 suppresses tumor metastasis by activating the HDAC8/ID2 pathway in hepatocellular carcinoma. *Cancer Letters*.

[15] Han D., Williams E., Cadenas E. (2001). Mitochondrial respiratory chain-dependent generation of superoxide anion and its release into the intermembrane space. *Biochemical Journal*.

[16] Janssen-Heininger Y. M. W., Mossman B. T., Heintz N. H. (2008). Redox-based regulation of signal transduction: principles, pitfalls, and promises. *Free Radical Biology and Medicine*.

[17] Terman A., Kurz T. (2013). Lysosomal iron, iron chelation, and cell death. *Antioxidants and Redox Signaling*.

[18] Kalyanaraman B., Cheng G., Hardy M., Ouari O., Bennett B., Zielonka J. (2018). Teaching the basics of reactive oxygen species and their relevance to cancer biology: mitochondrial reactive oxygen species detection, redox signaling, and targeted therapies. *Redox Biology*.

[19] Mancias J. D., Wang X., Gygi S. P., Harper J. W., Kimmelman A. C. (2014). Quantitative proteomics identifies NCOA4 as the cargo receptor mediating ferritinophagy. *Nature*.

[20] Huang T., Sun Y., Li Y. (2018). Growth inhibition of a novel iron chelator, DpdtC, against hepatoma carcinoma cell lines partly attributed to ferritinophagy-mediated lysosomal ROS generation. *Oxidative Medicine and Cellular Longevity*.

[21] Sun Y., Li C., Feng J. (2019). Ferritinophagic flux activation in CT26 cells contributed to EMT inhibition induced by a novel iron chelator, DpdtpA. *Oxidative Medicine and Cellular Longevity*.

[22] Hirschhorn T., Stockwell B. R. (2019). The development of the concept of ferroptosis. *Free Radical Biology and Medicine*.

[23] Zhang Z., Zhang L., Zhou L., Lei Y., Zhang Y., Huang C. (2019). Redox signaling and unfolded protein response coordinate cell fate decisions under ER stress. *Redox Biology*.

[24] Fang B. A., Kovačević Ž., Park K. C. (2014). Molecular functions of the iron-regulated metastasis suppressor, NDRG1, and its potential as a molecular target for cancer therapy. *Biochimica et Biophysica Acta (BBA) - Reviews on Cancer*.

[25] Ito H., Watari K., Shibata T. (2020). Bidirectional regulation between NDRG1 and GSK3*β* controls tumor growth and is targeted by differentiation inducing factor-1 in glioblastoma. *Cancer Research*.

[26] GO L. D. (2017). Role of lipid peroxidation, iron and ferritinophagy. *Biochimica et Biophysica Acta*.

[27] Li L. L., Li H., Li Y. L. (2021). Ferritinophagy-mediated ROS production contributed to proliferation inhibition, apoptosis and ferroptosis induction in action of mechanism of 2-pyridylhydrazone dithiocarbamate acetate. *Oxidative Medicine and Cellular Longevity*.

[28] Lu Q., Wang W. W., Zhang M. Z. (2019). ROS induces epithelial-mesenchymal transition via the TGF-*β*1/PI3K/Akt/mTOR pathway in diabetic nephropathy. *Experimental and Therapeutic Medicine*.

[29] Xu Z., Feng J., Li Y. (2020). The vicious cycle between ferritinophagy and ROS production triggered EMT inhibition of gastric cancer cells was through p53/AKT/mTor pathway. *Chemico-Biological Interactions*.

[30] Chen H.-T., Liu H., Mao M.-J. (2019). Crosstalk between autophagy and epithelial-mesenchymal transition and its application in cancer therapy. *Molecular Cancer*.

[31] GO L. D. (2017). Role of lipid peroxidation, iron and ferritinophagy. *Biochimica et Biophysica Acta*.

[32] Wenzel S. E., Tyurina Y. Y., Zhao J. (2017). PEBP1 wardens ferroptosis by enabling lipoxygenase generation of lipid death signals. *Cell*.

[33] Yang W. S., Stockwell B. R. (2016). Ferroptosis: death by lipid peroxidation. *Trends in Cell Biology*.

[34] van Belzen N., Dinjens W. N., Diesveld M. P. (1997). A novel gene which is up regulated during colon epithelial cell differentiation and down-regulated in colorectal neoplasms. *Laboratory Investigation*.

[35] Hu Z.-Y., Xie W.-B., Yang F. (2015). NDRG1 attenuates epithelial-mesenchymal transition of nasopharyngeal cancer cells via blocking Smad2 signaling. *Biochimica et Biophysica Acta - Molecular Basis of Disease*.

[36] Kurdistani S. K., Arizti P., Reimer C. L., Sugrue M. M, Aaronson S. A, Lee S. W (1998). Inhibition of tumor cell growth by RTP/rit42 and its responsiveness to p53 and DNA damage. *Cancer Research*.

[37] Menezes S. V., Fouani L., Huang M. L. H. (2019). The metastasis suppressor, NDRG1, attenuates oncogenic TGF-*β* and NF-*κ*B signaling to enhance membrane E-cadherin expression in pancreatic cancer cells. *Carcinogenesis*.

[38] Samatov T. R., Tonevitsky A. G., Schumacher U. (2013). Epithelial-mesenchymal transition: focus on metastatic cascade, alternative splicing, non-coding RNAs and modulating compounds. *Molecular Cancer*.

[39] Du B., Shim J. (2016). Targeting epithelial-mesenchymal transition (EMT) to overcome drug resistance in cancer. *Molecules*.

[40] Lee J.-Y., Kong G. (2016). Roles and epigenetic regulation of epithelial-mesenchymal transition and its transcription factors in cancer initiation and progression. *Cellular and Molecular Life Sciences*.

[41] Kaufhold S., Bonavida B. (2014). Central role of Snail1 in the regulation of EMT and resistance in cancer: a target for therapeutic intervention. *Journal of Experimental & Clinical Cancer Research*.

[42] Giannoni E., Parri M., Chiarugi P. (2012). EMT and oxidative stress: a bidirectional interplay affecting tumor malignancy. *Antioxidants and Redox Signaling*.

[43] Karicheva O., Rodriguez-Vargas J. M., Wadier N. (2016). PARP3 controls TGF*β* and ROS driven epithelial-to-mesenchymal transition and stemness by stimulating a TG2-Snail-E-cadherin axis. *Oncotarget*.

[44] Zhang K.-H., Tian H.-Y., Gao X. (2009). Ferritin heavy chain-mediated iron homeostasis and subsequent increased reactive oxygen species production are essential for epithelial-mesenchymal transition. *Cancer Research*.

[45] Guan D., Li C., Li Y. (2021). The DpdtbA induced EMT inhibition in gastric cancer cell lines was through ferritinophagy-mediated activation of p53 and PHD2/hif-1*α* pathway. *Journal of Inorganic Biochemistry*.

[46] Dixon S. J., Lemberg K. M., Lamprecht M. R. (2012). Ferroptosis: an iron-dependent form of nonapoptotic cell death. *Cell*.

[47] Su L. J., Zhang J. H., Gomez H. (2019). Reactive oxygen species-induced lipid peroxidation in apoptosis, autophagy, and ferroptosis. *Oxidative Medicine and Cellular Longevity*.

[48] Sun L., Dong H., Zhang W. (2021). Lipid peroxidation, GSH depletion, and SLC7A11 inhibition are common causes of EMT and ferroptosis in A549 cells, but different in specific mechanisms. *DNA and Cell Biology*.

[49] Li A., Zhu X., Wang C. (2019). Upregulation of NDRG1 predicts poor outcome and facilitates disease progression by influencing the EMT process in bladder cancer. *Scientific Reports*.

[50] Chen Z., Sun J., Li T. (2018). Iron chelator-induced up-regulation of Ndrg1 inhibits proliferation and EMT process by targeting Wnt/*β*-catenin pathway in colon cancer cells. *Biochemical and Biophysical Research Communications*.

