# Supplementary material for: Ferritinophagic Flux Was a Driving Force in Determination of Status of EMT, Ferroptosis, and NDRG1 Activation in Action of Mechanism of 2-Pyridylhydrazone Dithiocarbamate S-Acetic Acid
Source: J Oncol. 2021 Dec 7;2021:3015710. doi: 10.1155/2021/3015710 (PMC8670909; doi:10.1155/2021/3015710)
Supplement: Supplementary Materials — Figure S1. The effect of PdtaA on morphology of HepG2 cell. (a) DMSO (70%). (b) PdtaA. Arrowheads are the cells that underwent morphologic change. Objective size: 20 ×10. Figure S2. PdtaA induced a downregulation of ferroptosis-related proteins. (a) Western blotting analysis and (b) quantitative analysis derived from (A). ##,∗∗P < 0.05; ∗∗∗, ###p < 0.01 vs. control. Figure S3. Chloroquine attenuated the action of PdtaA on the regulatory effect on ferroptosis-related proteins. (a) Western blotting analysis and (b) quantitative analysis derived from (A). ##, ∗∗, $$P < 0.05; ∗∗∗p < 0.01 vs. control. Figure S4. PdtaA treatment results in alteration in lipid peroxidation. ∗∗∗, ###P < 0.01 vs. control. Figure S5. PdtaA treatment results in an upregulation of NDRG1. (a) Western blotting analysis and (b) quantitative analysis derived from (A). ∗∗P < 0.05; ∗∗∗p < 0.01 vs. control. Figure S6. Activation of NCOA4 and NDRG1 involved PdtaA-induced ferroptosis. The HepG2 cells treated with either siRNA-mate or siRNA-NCOA4, followed by PdtaA treatment. The lipid peroxidation was measured based on the method described previously. The molar absorptivity of the ferric thiocyanate complex expressed per mol of LOOH was determined to be 58,440 M−1 cm−1 [2]. ##P < 0.05 vs. control; ∗∗∗p < 0.01 vs. control. [file 3015710.f1.docx]

**Ferritinophagic flux was a driving force in determination of status of EMT, ferroptosis, and NDRG1 activation in action of mechanism of 2-pyridylhydrazone dithiocarbamate s-acetic acid**

Hao Li^1†^, Wei Zhou^2†^, Huiping Wei^2†^, Longlong Li^1^, Xu Wang^2^, Yongli Li^2*^, Shaoshan Li^1*^ and Changzheng Li^3,4*^

^1^Department of Surgery, the Third Affiliated Hospital of Xinxiang Medical University, Xinxiang, Henan, P. R. China, 453003 ;

^2^Department of Histology and Embryology, Sanquan College of Xinxiang Medical University, Xinxiang, Henan, P. R. China, 453003

^3^College of Pharmacy, Sanquan College of Xinxiang Medical University, Xinxiang, Henan, P. R. China, 453003;

^4^College of Basic Medical Science, Xinxiang Medical University, Xinxiang, Henan, P. R. China, 453003;

;

**Supplementary Materials**

**PdtaA induced morphologic alteration**

The structure of 2-pyridylhydrazone dithiocarbamate acetate (PdtaA) was shown in insert of Fig. S1B, PdtaA exhibited significant antitumor activity against HepG2 cell at micromole level in our previous study [1], the morphologic alteration after the treatment of the agent was observed. Fig. S1 showed that the spindle cells in 70% DMSO (Fig. S1A) were changed to round cells (arrowheads in Fig. S1B) upon the cells exposure to the agent for 24 h, this cytoskeleton reorganization promoted up to consider that whether there was an occurrence of epithelial-to-mesenchymal transition (EMT) during the agent treatment.

**
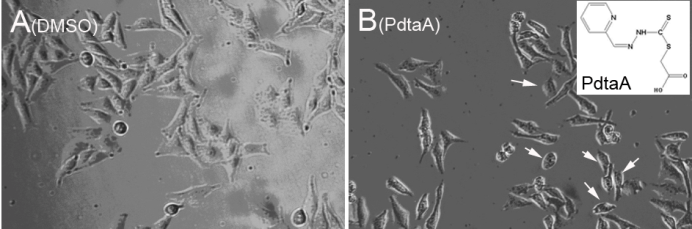
**

Figure S1: The effect of PdtaA on morphology of HepG2 cell. (A) DMSO (70%); (B) PdtaA. Arrowheads were the cells that underwent morphologic change. Objective size: 20 ×10.

**PdtaA induced an occurrence of ferroptosis**

PdtaA-induced EMT was ROS-dependent, thus the feritinophagy-mediated ROS production might cause ferroptosis. To this end, the levels of Gpx4 and xCT were investigated for inhibition of system xc^−^ promotes the iron-mediated accumulation of lipid peroxidation end products and ultimately induces ferroptosis [2]. As shown in Fig. S2A, PdtaA treatment resulted in a significant decrease in both Gpx4 and xCT, the quantitative analysis was presented in Fig. S2B. Those hinted that ferroptosis may be involved in the action of mechanism of PdtaA.

**
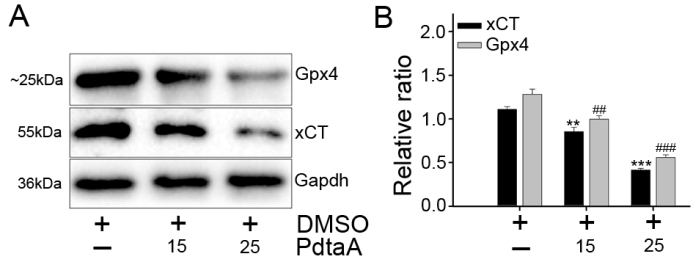
**

**Figure S2**. PdtaA induced a downregulation of ferroptosis-related proteins. (A) western blotting analysis and (B) quantitative analysis derived from (A). ^##.**^P<0.05, ^***,###^P<0.01 vs control.

**Inhibition of autophagy attenuated the ability of PdtaA in ferroptosis induction**

To support that ferritinophagic flux was a driving force in ferroptosis induction, an autophagy inhibitor, chloroquine was used to confirm that the action of PdtaA was an autophagy dependent. As shown in Fig. S3, PdtaA treatment resulted in a significant increase of ferritinophagic flux, a markedly decrease in both xCT and Gpx4, however, addition of chloroquine could resist the regulatory effect of PdtaA on ferroptosis, supporting that the ferritinophagic flux was a driving force in ferroptosis induction in action of mechanism of PdtaA.


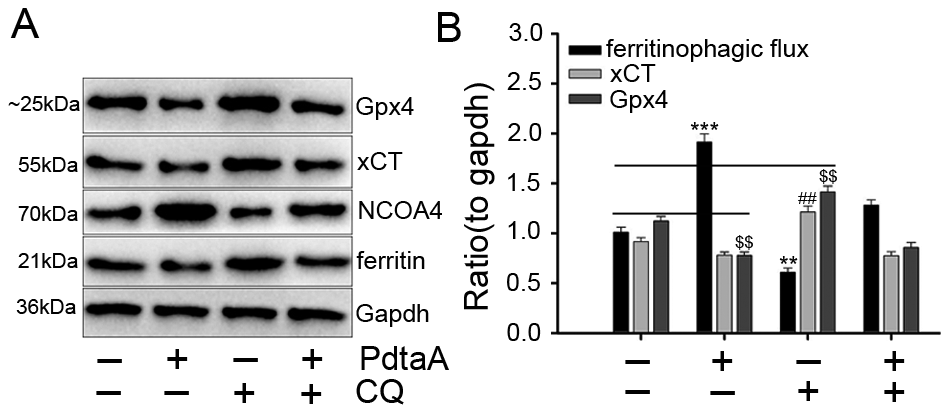


**Figure S3**. Chloroquine attenuated the action of PdtaA on regulatory effect on ferroptosis-related proteins. (A) western blotting analysis and (B) quantitative analysis was derived from (A). ^##.**,$$^P<0.05, ^***^P<0.01 vs control

**PdtaA treatment resulted in lipid peroxidation**

Lipid peroxidation analysis was performed based on spectrophotometry, in which the ferrous ion is oxidized by lipid hydroperoxides to the ferric ion and subsequently reacts with thiocyanate to form a colored complex [3]. The assay was performed according to the previously described protocol [4]. Briefly, the trypsinized cells were collected and treated with the PdtaA for 24 h. The supernatant was removed by centrifugation and washed with PBS. The peroxidized lipids were extracted using deoxygenated CHCl_3_/MeOH (2:1, v/v mixture; 1,000 μl), and the lipids were transferred to a 5 ml volumetric flask, which contained 100 μl of ferrous sulfate (0.2 M HCl) and 100 μl of 3% deoxygenated thiocyanate (methanol) for 60 min. Finally, deoxygenated CHCl_3_/MeOH solvents were added to the given volume. The absorbance at 500 nm was measured using a UV-2450 spectrophotometer (Shimadzu Corporation). The molar absorptivity of the ferric thiocyanate complex expressed per mol of LOOH was determined to be 58,440 M^-1^ cm^-1^ [3].

As shown in Fig. S4, the level of peroxidized lipids was significantly increased and addition of ferroptosis inhibitor, ferostatin-1 attenuated the lipids peroxidation. In addition, addition of NAC also led to a decrease of lipid peroxidation. Those indicated that that ROS production resulted in lipid peroxidation and ferroptosis induction in action of mechanism of PdtaA.

**
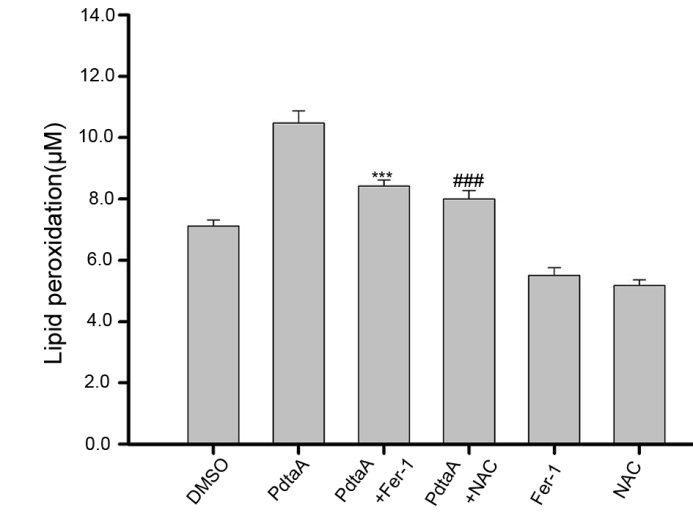
**

**Figure S4**. PdtaA treatment results in alteration in lipid peroxidation. ^***,###^P<0.01 vs control.

**PdtaA treatment resulted in upregulation of NDRG1**

It has been revealed that NDRG1 responds the treatment of iron chelator and associates with EMT inhibition [4], PdtaA as an iron chelator, the NDRG1 activation might also involve the action of mechanism of the agent. To this end, the level of NDRG1 was assayed via western blotting. As shown in Fig. S5A, the PdtaA treatment resulted in a significant increase of NDRG1, in consistent with that reported previously [5]. The quantitative analysis was presented in Fig. S5B, clearly, NDRG1 activation was in a concentration-dependent manner.

**
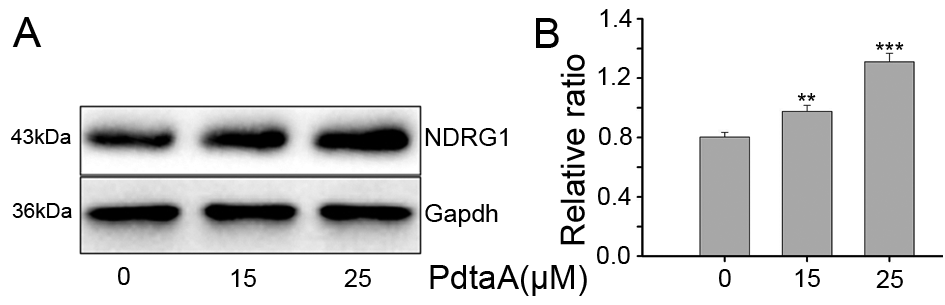
**

**Figure S5.** PdtaA treatment results in an upregulation of NDRG1. (A) western blotting analysis and (B) quantitative analysis derived from (A). ^**^P<0.05, ^***^P<0.01 vs control.

**Knockdown of NCOA4 and NDRG1 by siRNA attenuated the lipid peroxidation induced by PdtaA**

Since PdtaA could induce ferritinophagy, trigger ROS production through Fenton reaction, and lead to an occurrence of ferroptosis. As a stress responder, NDRG1 may respond to the alteration in redox environment. How NDRG1 responded to ferroptosis needed to be determined. To corroborate the role of NDRG1 in ferroptosis induction, the NDRG1 was genetically knocked down by small interfering RNA. As shown in Fig. S6, knockdown of both NCOA4 and NDRG1 caused attenuation of lipid peroxidation, supporting that PdtaA induced ferroptosis correlated with ferritinophagy and activation of NDRG1.


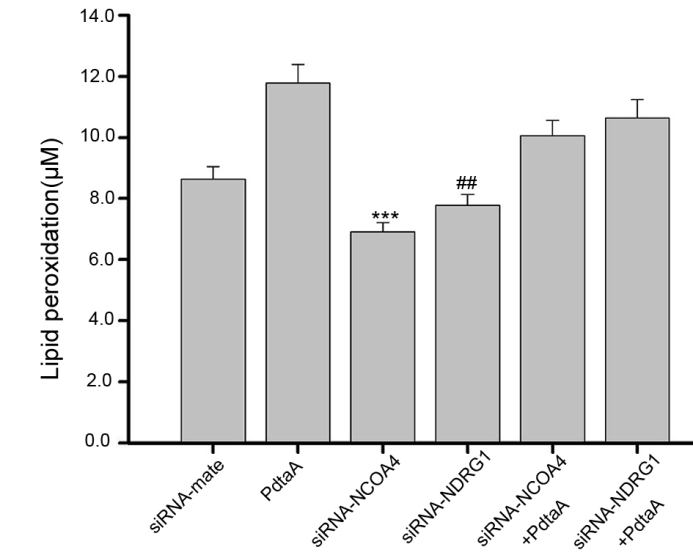


**Figure S6**. Activation of NCOA4 and NDRG1 involved PdtaA induced ferroptosis. The HepG2 cells treated with either siRNA-mate or siRNA-NCOA4, followed by PdtaA treatment. The lipid peroxidation was measured based on the method described previously. The molar absorptivity of the ferric thiocyanate complex expressed per mol of LOOH was determined to be 58,440 M^-1^ cm^-1^[2]. ^##^P<0.05 vs control, ^***^P<0.01 vs control.

**References**

1. L.L. Li, H. Li, Y. L. Li, et al., “Ferritinophagy-mediated ROS production contributed to proliferation inhibition, apoptosis and ferroptosis induction in action of mechanism of 2-pyridylhydrazone dithiocarbamate acetate,” *Oxidative Medicine and Cellular Longevity*, vol. 2021, ID5594059, 2021.
2. D.H. Kim, W.D. Kim, S.K. Kim, D.H. Moon, S.J. Lee, “TGF-β1-mediated repression of SLC7A11 drives vulnerability to GPX4 inhibition in hepatocellular carcinoma cells,” Cell Death & Disease, vol. 11, no. 5, pp. 406, 2020.
3. A.C. Gasparovic, M. Jaganjac, B. Mihaljevic, et al., “Assays for the measurement of lipid peroxidation,” *Methods in Molecular Biology*, vol. 965, pp. 283-296, 2013.
4. J. Riemer, H. H. Hoepken, H. Czerwinsk, et al., “Colorimetric ferrozine-based assay for the quantitation of iron in cultured cells,” *Analytical Biochemistry*, vol. 331, no. 2, pp. 370-375, 2004
5. Z. Chen, J. Sun, T. Li, et al., “Iron chelator-induced up-regulation of Ndrg1 inhibits proliferation and EMT process by targeting Wnt/β-catenin pathway in colon cancer cells,” *Biochemical and Biophysical Research Communications*, vol. 506, no. 1, pp. 114–121, 2018.
